# Development and Application of a 40 K Liquid Capture Chip for Beef Cattle

**DOI:** 10.3390/ani15091346

**Published:** 2025-05-07

**Authors:** Qing Liu, Liangyu Shi, Pu Zhang, Bo Yu, Chenhui Liu, Min Xiang, Shuilian Li, Lei Liu, Lei Cheng, Hongbo Chen

**Affiliations:** 1 Laboratory of Genetic Breeding, Reproduction and Precision Livestock Farming & Hubei Provincial Center of Technology Innovation for Domestic Animal Breeding, School of Animal Science and Nutritional Engineering, Wuhan Polytechnic University, Wuhan 430023, China; liuqing@whpu.edu.cn (Q.L.); liangyu_shi@whpu.edu.cn (L.S.); z15515092327@163.com (P.Z.); wonderfish@whpu.edu.cn (B.Y.); 2 Institute of Animal Science and Veterinary Medicine, Wuhan Academy of Agricultural Sciences, Wuhan 430208, China; lchhcl890621@sina.com (C.L.); xm11609@126.com (M.X.); 3 Hubei Jinchu Husbandry Co., Ltd., Wuhan 430014, China; m17739749922@163.com (S.L.); lance521@163.com (L.L.)

**Keywords:** beef cattle, chip development, liquid chip, runs of homozygosity, single-nucleotide polymorphism loci

## Abstract

The availability of genome sequences and single-nucleotide polymorphism (SNP) chips allows us to investigate the various genomic characteristics of species by exploring genetic diversity to aid genetic selection. The SNP chip is a cost-effective genotyping platform essential for molecular breeding of livestock. In this study, we developed a liquid SNP capture chip suitable for five Hubei (China) indigenous beef cattle breeds based on whole-genome sequencing datasets. The panel consisted of 42,686 SNPs (~40 K). These SNPs were evenly distributed on each bovine chromosome, with the majority of SNPs having minor allele frequencies >0.05 and located within intergenic regions. The performance evaluation of this SNP chip panel was proceeded by genotyping 200 individuals, revealing that this panel has a high SNP call rate of 99.48%. The SNP chip panel was further used to examine the population structure of a beef cattle population with 205 individuals and demonstrated the ability to differentiate between foreign and indigenous cattle breeds. The SNP chip was also used to determine the runs of homozygosity (ROH) within a local Hubei beef cattle population of 195 individuals. We identified 2547 ROH and several genes associated with economically important traits in the study population. Our findings demonstrate that this chip not only contributes to the understanding of the genetic characteristics of local beef cattle breeds but also provides valuable genetic information for future breeding programs, thereby improving their production efficiency and economic value.

## Simple Summary

The evaluation and utilization of local germplasm resources still pose limitations to the efficient and sustainable development of the beef cattle industry in China. With the increasing amount of sequencing data, the development of single-nucleotide polymorphism (SNP) chips has increased, enabling the evaluation, development, and utilization of local germplasm resources. Here, we developed a 40 K liquid SNP capture chip and evaluated its performance in genotyping, efficacy in population genetic studies, and practicality in identifying runs of homozygosity (ROH). These findings demonstrate that this chip not only contributes to the understanding of the genetic characteristics of local beef cattle breeds but also provides valuable genetic information for future breeding programs, thereby improving their production efficiency and economic value.

## 1. Introduction

Cattle are the most widely distributed species of livestock and provide humans with products such as meat, milk, leather, and labor [1]. In China, the beef cattle industry is growing rapidly owing to growing consumer demand and government-supported policies. China also has abundant beef cattle breed resources, among which there are five well-known indigenous beef cattle breeds in Hubei Province: Enshi (ES), Huangpi (HP), Yunba (YB), Yiling (YL), and Zaobei (ZB). These local beef cattle breeds exhibit strong limbs, well-proportioned body shape, heat resistance, labor tolerance, rough feeding resistance, delicious meat quality, and rich nutritional value because of their successful adaption to subtropical climate and environmental conditions. However, the genetic potential of these beef cattle breeds has not been fully explored. Consequently, it is imperative to explore effective strategies for the conservation and utilization of genetic resources in indigenous beef cattle in Hubei.

Advances in genomic technologies have revolutionized the livestock industry, particularly the use of single-nucleotide polymorphisms (SNPs), which have facilitated the exploration of genetic characteristics and the improvement of important economic traits in livestock species [2,3,4]. SNP chips have been widely used in various genomic studies in animal husbandry [5,6,7]. As large-scale genotyping platforms, SNP chips have been developed and used for genomic studies in various livestock species, including cattle [8], sheep [9], goats [10], pigs [11], and buffalo [12], which has accelerated genetic progress in this species. These chip data are mainly used for the analysis of genetic diversity [13], population genetics [14], selective sweeps [15], quantitative trait site (QTL) mapping [16], genome-wide association studies (GWAS) [17], runs of homozygosity (ROH) [18], and genomic selection (GS) [19]. SNP chip technology plays an increasingly prominent role in animal husbandry, which not only accelerates the process of genetic improvement but also improves the accuracy and efficiency of trait improvement.

SNP chip technology can be categorized into two main types: solid-phase and liquid chips. Traditional solid-phase chips rely primarily on complementary hybridization between probes and DNA sequences and are analyzed through the fluorescent signals of labeled probes. This has some disadvantages, such as high typing costs and challenges in personalized development. Compared to traditional solid-phase chip technology, liquid chip technology, also known as genotyping by target sequencing (GBTS) liquid chips, combines the advantages of traditional solid-phase chips and next-generation sequencing technology with features such as high detection accuracy, cost-effectiveness, flexible design, and the ability to incorporate new sites [20,21]. GBTS has been widely applied to livestock species, including chickens [22], pigs [23], sheep [24], goats [25], and cattle [1]. GBTS has also been utilized in various genomic studies, such as the Cattle110K capture chip adapted for genomic prediction [21], liquid chips suitable for GWAS [24,25], and 10 K cGPS liquid chips used for research and protection of Hainan black goat germplasm resources [26].

To date, no liquid chip has been specifically designed for Hubei indigenous beef cattle. This study presents the development and application of a 40K liquid capture chip. We validated its performance by conducting population genetic studies on 205 individuals and identifying the ROH in 195 individuals from four local cattle breeds. Our findings suggest that this chip is a powerful tool for genetic breeding and genomic research in beef cattle, specifically for local Hubei breeds.

## 2. Materials and Methods

### 2.1. Animals and Sampling

A total of 98 beef cattle were obtained from five local breeds (18 ZB, 20 YL, 18 YB, 14 ES, and 28 HP) in Hubei province, China. The animals consisted of bulls and cows, and ear tissue samples were collected. Genomic DNA was isolated from each sample using the standard phenol–chloroform method. The extracted DNA was quantified using a Qubit fluorometer and the quality was assessed using a Nanodrop spectrophotometer. Sequencing libraries were constructed using a GenoBaits^®^ DNA Library Prep Kit for Illumina following the manufacturer’s instructions. The libraries were sequenced on an Illumina HiSeq X Ten platform with 150 bp paired-end sequencing. Furthermore, 240 whole-genome sequencing (WGS) datasets from the public NCBI database were downloaded, representing four foreign breeds of beef cattle: Simmental (N = 67), Charolais (N = 45), Angus (N = 38), and Limousin (N = 90).

### 2.2. SNP Discovery

All WGS raw reads were checked for sequencing quality using Trimmonmatic v0.39 software [27]. All clean reads were aligned to the bovine reference genome (version: *ARS-UCD1.2*) using BWA-MEN v2.2.1 [28] with default settings. Furthermore, a Genome Analysis Toolkit (GATK, v4.2.0.0) [29] was used to perform variant detection based on the following filtering parameters: quality by depth (QD) < 2.0, QUAL < 30.0, symmetric odds ratio (SOR) > 3.0, Fisher strand (FS) > 60.0, root mean square of mapping quality (MQ) < 40.0, MQRankSum< −12.5, and ReadPosRankSum < −8.0. PLINK v1.9 [30] software was used to further extract variants with SNPs with missing rates < 0.1 and minor allele frequencies (MAF) < 0.05. A total of 1,210,156 SNPs and 264 individuals were used for subsequent analyses. Detailed information on the WGS dataset for 98 beef cattle is presented in Supplementary Table S1.

### 2.3. Development of Liquid Capture Chip Panel

The study design and workflow of the 40 K liquid capture chip for Hubei indigenous beef cattle are shown in Supplementary Figure S1. Here, several hierarchical methods were used to select SNP loci for the chip panel. Overall, we collected SNP loci for different biological purposes in three stages. In the first stage, we used TRES [31] software to identify breed-specific sites for five Hubei indigenous breeds and four foreign breeds. Three methods, including Wright’s Fst, detla, and informativeness for assignment (In), implemented in TRES software, were used to analyze the genotypic dataset. After synthesizing the differences between comparison groups, SNP loci with the top 1000, top 2000, top 3000, top 4000, and top 5000 were selected and used for principal component analysis (PCA). Finally, the most representative SNP loci were selected as candidate breed-specific sites based on grouping results. In the second stage, we collected and analyzed published SNP loci from a previous study [32]. These loci were designated trait-related sites in the chip panel and involved bovine genetic defects and economically important traits such as growth, carcass, beef quality, milk production, reproduction, and body shape. In the third stage, we selected common sites from five widely and commercially available bovine breeding chips, including 50 K, 60 K, 80 K, 777 K, and 150 K bovine SNP chips.

All the aforementioned SNP loci were merged with the previous reports described by Liu et al. [33]. In this study, we assigned priority levels to SNP sites based on their identification at different stages. The SNP sites from the first and second stages were designated priority 1, the common sites from the third stage priority 2, and the polymorphic sites (MAF > 0.2) identified through WGS data priority 3. Subsequently, the optimization selection process was executed using a window-based approach for priority-filling analysis. Bovine chromosomes were segmented using a window of 50 K. The frequencies of the candidate SNP loci were quantified within each window across all chromosomes. According to the number of SNPs in each window, we selected the candidate sites for the chip panel using the following screening method. If there were only one SNP in a window, the locus was retained. If there were two or more SNPs in a window, only two loci in this window would be retained, which enabled the SNPs to be evenly distributed in this window based on the following formula:

$${SD}^{2}=\frac{{\left(S-\bar{X}\right)}^{2}+{\left(N_{i}-\bar{X}\right)}^{2}+{\left(N_{j}-\bar{X}\right)}^{2}+{\left(E-\bar{X}\right)}^{2}}{4}$$

where *S* and *E* are the start position and end position of the window, respectively, and $N_{i}$ and $N_{j}$ are the target SNP positions in the window. The SNPs $N_{i}$ and $N_{j}$, which can minimize the SD^2^, were reserved.

If there were no SNP in the window, supplementation of the filtered loci with no selection signature was required. Then, according to the additional information from these loci, the SNP loci were screened in the order of SNP priority. The loci with the highest priority were chosen as the candidate loci. The preparation/development of the GBTS 40K genotyping test kit was performed by MolBreeding Biotech Ltd. (Shijiazhuang, China).

### 2.4. Validation of the 40K Chip Panel

A total of 200 additional individuals from five local Hubei cattle breeds (33 ES, 39 HP, 52 YL, 71 ZB, and 5 YB) were genotyped using the 40K SNP chip panel. To gain a better understanding of panel performance, we performed a comparative analysis of SNP call and missingness rates between different sexes and breeds. The *t*-test was used to determine the statistical significance between the contrasting groups. Statistical significance was set at *p* < 0.05.

### 2.5. Population Genetic Analysis

We genotyped 195 individuals (33 ES, 39 HP, 52 YL, and 71 ZB) representing four beef cattle breeds using the 40K liquid capture chip. In addition, we collected SNP loci from 10 individuals representing Simmental (SM; N = 5) and Charolais (CL; N = 5) breeds. In total, 205 individuals and 35 193 SNPs were used for subsequent analysis. To investigate the genetic relationships among the studied populations, PLINK v1.9 software [30] was used to perform PCA. The “ggplot2” R package was used to visualize the first two principal components (PCs). Subsequently, the VCF2Dis v1.53 software [34] was used to calculate the p-distance matrix, which was used to construct the phylogenetic tree. The phylogenetic tree was visualized using iTOL online software. Admixture v1.3.0 software [35] was used to estimate the breed composition of the tested populations. Furthermore, to understand the genome-wide linkage disequilibrium (LD) of local Hubei beef cattle breeds, we performed genome-wide LDD decay (LDD) within breed groups using PopLDdecay v3.43 software [36].

### 2.6. Identification of Runs of Homozygosity

PLINK v1.90 software [30] with the sliding-window approach was used to determine the ROH within the local Hubei beef cattle population. The parameters were set as follows: (i) the minimum length of the run was 1 Mb; (ii) the maximum gap between consecutive SNP to still be considered a potential run was 1 Mb; (iii) the minimum SNP density was set to 1 SNP every 100 kb; (iv) the size of the sliding window was 50 SNPs; (v) one heterozygous genotype per window was allowed; (vi) the threshold of overlapping windows of the same state (homozygous or heterozygous) to call an SNP in a run was set to 0.01; and (vii) the number of SNPs per ROH was calculated using the following equation:

$$L=\frac{{log}_{e}\frac{\alpha }{n_{s}n_{i}}}{{log}_{e}\left(1-het\right)},$$

where $n_{s}$ is the number of genotyped SNPs per animal, $n_{i}$ is the number of genotyped animals, α is 0.05, and het is the mean heterozygosity across all SNPs.

### 2.7. Detection and Analyses of Common Runs of Homozygosity

To identify genomic regions characterized by a high frequency of ROH occurrence, the number of times each SNP occurred in ROH was calculated across individuals. The top 1% of the highest occurrence values was chosen as the threshold, as suggested in a previous study [37], in which the identified genomic regions were classified as “ROH islands” in a given population. The frequency of ROH was plotted against their physical position in the bovine genome (*ARS-UCD1.2*). Genes within each ROH island were extracted from bovine genome annotation using Bedtools v2.28 software [38]. These genes were also used to identify overlapping QTL described in the Cattle Quantitative Traits Locus Database (Cattle QTLdb) (ver.54; https://www.animalgenome.org/cgi-bin/QTLdb/BT/index (accessed on 12 December 2024)). The STRING database (ver.12.0; https://string-db.org (accessed on 17 December 2024)) was used to predict protein–protein interaction (PPI) relationships among the annotated genes. KOBAS software [39] was used to identify significantly enriched Gene Ontology (GO) terms and Kyoto Encyclopedia of Genes and Genomes (KEGG) pathways (adjusted *p*-value < 0.05) of the genes located in ROH regions.

## 3. Results

### 3.1. Characterization of the Customed 40K Chip Panel

**Figure 1:**
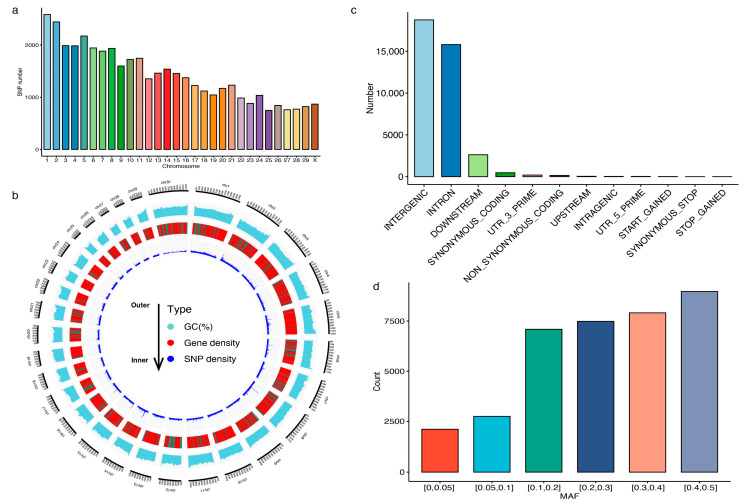
Characterization of the 40K liquid capture chip. (**a**) Density distribution of single-nucleotide polymorphisms (SNPs) in bovine chromosomes. (**b**) Circos plot showing the density of SNPs and genes on each chromosome. Red indicates a gene count of 50 or more in a 1 Mb window, dark blue indicates a gene count of 50 or more over 35 in a 1 Mb window, and brown indicates a gene count of 1 in a 1 Mb window. (**c**) Distribution patterns of SNPs over different regions of the bovine genome. (**d**) Number distribution of SNPs based on the MAF categories.

To accommodate more potential functional sites on the chip, we selected three types of sites from different sources: breed-specific, trait-related, and common sites. A total of 42,686 SNPs were included in the 40K chip panel, with 4000 sites being breed-specific, 8193 sites being trait-related SNP loci, 19,459 sites being common sites, and 11,034 being polymorphic sites (MAF > 0.2) from WGS data (Supplementary Figure S1). The SNPs on the chip panel were evenly distributed on each bovine chromosome (Figure 1a). The number of SNP loci in the bovine chromosome varied with length, with chromosome 1 having the highest SNP number (N = 2585) and chromosome 25 having the lowest SNP number (N = 747). Notably, GC content was evenly distributed on each chromosome of the bovine genome, and the genes tended to be distributed on both sides of the chromosome (Figure 1b). In particular, the SNP loci in this chip exhibited higher peaks on both sides of the chromosome, suggesting that the SNP loci in this chip may be related to gene location. SNP annotation analysis showed that most SNP loci on the chip were mainly distributed in the intergenic region, followed by the intron and downstream regions (Figure 1c). Moreover, the MAF value of SNP loci on the chip was generally higher than 0.05 (Figure 1d), indicating that the chip design had a reasonable amount of genetic diversity.

### 3.2. Genotyping Performance of the 40K Chip Panel

**Figure 2:**
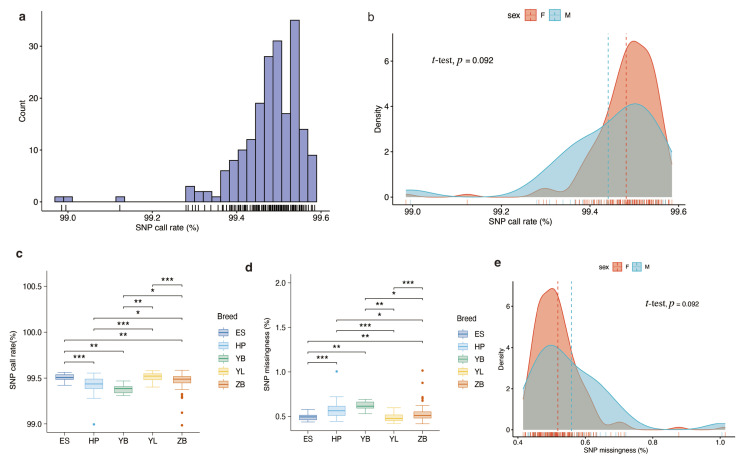
Genotyping performance of the liquid SNP chip panel. (**a**) Distribution of SNP call rates in the study population. (**b**) Comparative analysis of SNP call rates between males and females. (**c**) Comparative analysis of SNP call rates among the different breeds. (**d**) Comparative analysis of SNP missingness rates among the different breeds. (**e**) Comparative analysis of SNP missingness rates between males and females. *, ** and *** indicate *p* < 0.05, *p* < 0.01, and *p* < 0.001, respectively.

To validate the genotyping performance of the 40 K chip panel, 200 individuals from five local Hubei cattle breeds were genotyped. In total, 40,477 SNPs (94.83% of all SNP loci in the panel) were detected in the study population. The average SNP call rate was approximately 99.48% (Figure 2a). We observed a higher SNP call rate in females than in males, but no significant difference (*p*-value = 0.092) was found between them (Figure 2b). In addition, we found that the SNP call rate varied among the breeds (Figure 2c). All studied breeds exhibited high SNP call rates (>99%), with three breeds (ES, YL, and ZB) demonstrating SNP call rates exceeding 99.5%. Moreover, a lower SNP missingness rate was observed in the studied breeds, with a mean SNP missingness rate of less than 1%. It is noteworthy that the SNP missingness rates among the comparison breeds exhibited statistically significant differences (*p*-value < 0.05; Figure 2d), whereas no significant difference was observed between males and females (*p*-value = 0.092; Figure 2e).

### 3.3. Population Structure Analysis Based on the Chip Data

**Figure 3:**
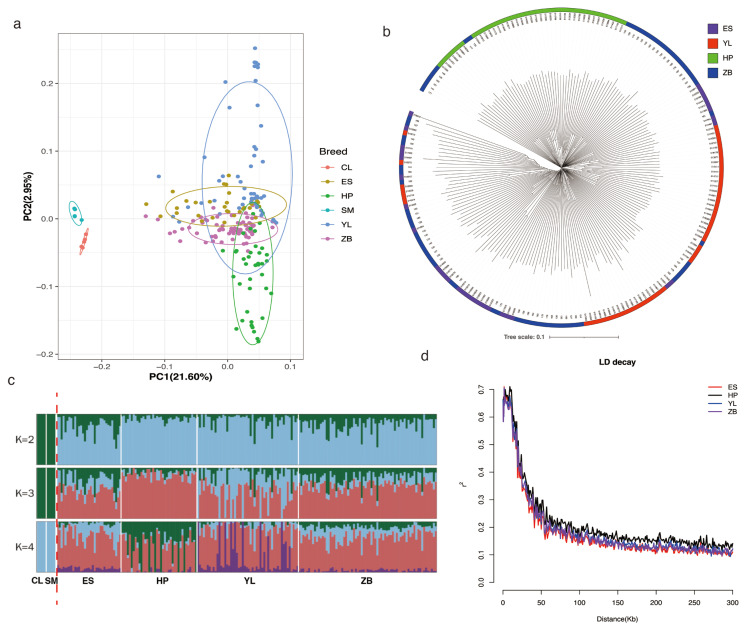
Population genetic structure and phylogenetic tree analyses. (**a**) Principal component analysis of 205 individuals. (**b**) Phylogenetic trees of 195 local cattle in Hubei. (**c**) Population ancestry composition analysis of 205 individuals. (**d**) LD decay map of 4 local cattle herds in Hubei.

To evaluate the efficacy of the chip utilized in population genetic studies, we examined breed differentiation among the studied populations based on SNP data, encompassing 205 individuals from six beef cattle breeds. PCA revealed that individuals from each breed could be grouped into two distinct groups (Figure 3a). PC1 accounted for 21.60% of the total variation and separated the foreign breeds (CL and SM) from the local Hubei breeds. PC2 accounted for 2.95% of the explainable variance, which distinctly differentiated the two breeds (HP and YL). Phylogenetic analysis further supported the PCA results, with HP and YL being relatively independent clusters (Figure 3b). In addition, phylogenetic tree analysis revealed that a small subset of individuals exhibited genetic admixture among the four local beef cattle breeds in Hubei. Furthermore, admixture results for K = 2 to K = 4 are shown in Figure 3c, and show that K = 3 represented the most appropriate population number for the present dataset. These results suggest that these animals can be divided into two groups: foreign breeds (CL and SM) and local Hubei breeds. Notably, these local breeds can be inferred to be crossbreeds because they are adulterated with the bloodlines of Charolais and Simental cattle.

Given the significance of LD decay in elucidating the population recombination history, we conducted an LDD analysis for local Hubei breeds using PopLDdecay software. The results showed that the overall estimated LD differed between breeds (Figure 3d). As expected, the LD estimated between pairwise SNPs generally declined with increasing physical distance. The LDD in the ES breed declined faster than in other breeds with increasing distance, whereas the HP breed showed the slowest decline. The average distance when LDD decreased to a value of 0.2 was approximately 50 Kb for the local Hubei breeds.

### 3.4. Runs of Homozygosity Analysis Based on Chip Data

**Figure 4:**
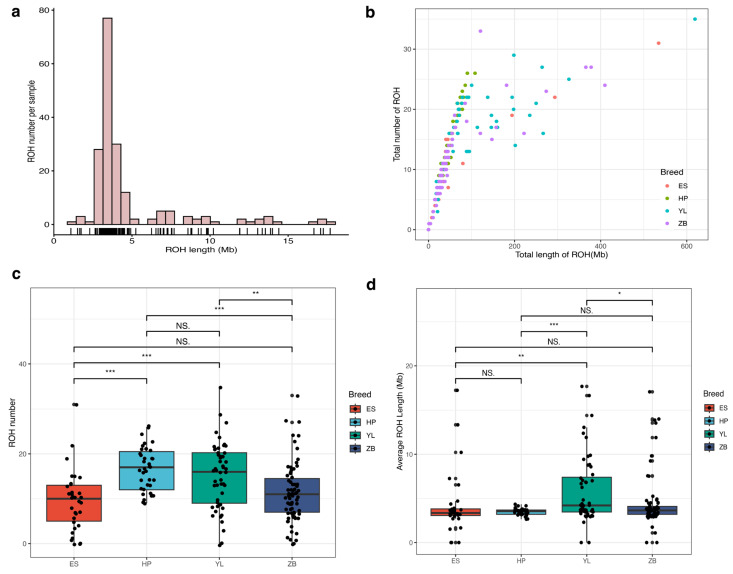
Genomic runs of homozygosity (ROH) patterns in local Hubei cattle breeds. (**a**) Length distribution of ROH per sample. X-axis represents the length of ROH (Mb) using a base-10 log scale. (**b**) Relationship between total ROH length and ROH coverage in the studied population. (**c**) Box plot displaying ROH number distribution among breeds. (**d**) Box plot displaying the distribution of average ROH length among breeds. *, ** and *** indicate *p* < 0.05, *p* < 0.01, and *p* < 0.001, respectively. NS. indicates no significant, *p* > 0.05.

To further evaluate the practicality of the chip in identifying ROH, we performed an ROH analysis based on chip data from four local Hubei breeds. In total, 2547 ROH were identified in 195 individuals. Figure 4a shows the distribution of ROH lengths per individual. The average ROH size varied considerably from 1.09 to 17.69 Mb, with an average size of 4.60 Mb across all autosomes. We also investigated the relationship between the total number of ROH and total length of the genome covered by ROH. The results revealed a significant difference in the aforementioned relationship in the local Hubei cattle population (Figure 4b). Among these populations, both ROH length and coverage were higher in the ES and HP breeds. Conversely, the ROH length and ROH coverage of the YL and ZB cattle populations demonstrated more pronounced differentiation. Furthermore, a higher ROH number was identified in the HP and YL populations than in the other populations (Figure 4c). The number of ROH per animal varied from 1 in ES/ZB to 35 in YL cattle. Notably, the mean ROH length was higher in the YL population than in other populations (Figure 4d).

### 3.5. Runs of Homozygosity Involved in Economically Important Traits

**Figure 5:**
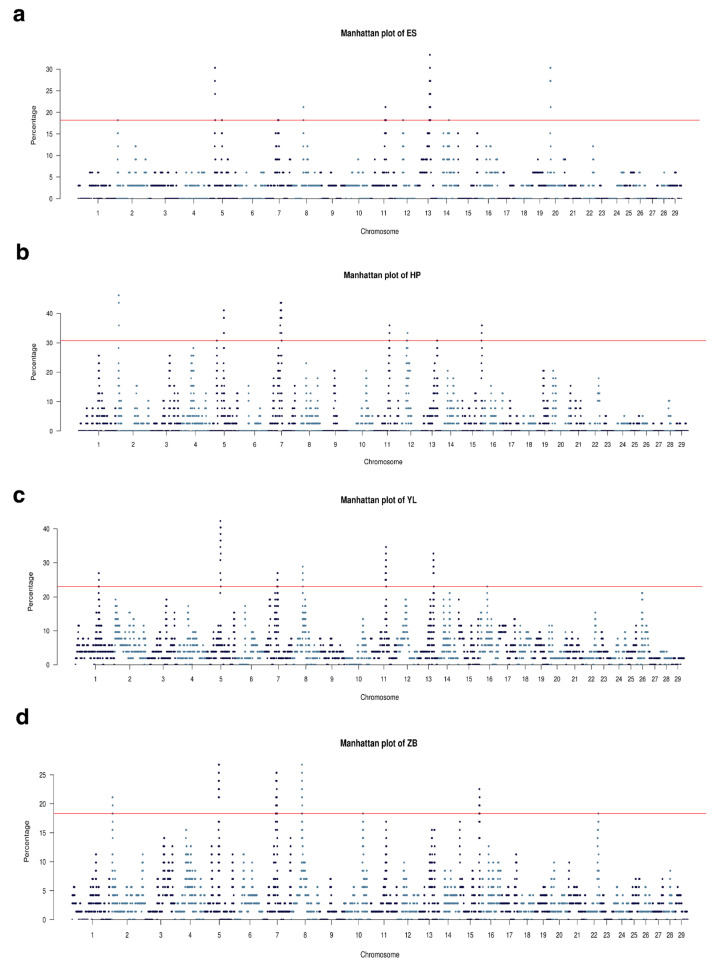
Manhattan plot of the percentage of SNPs in the ROH of ES (**a**), HP (**b**), YL (**c**), and ZB (**d**) cattle.

To investigate the effect of selection in local Hubei breeds, we analyzed the occurrence of ROH across the genome. In this study, the frequency of an SNP locus (%) within the identified ROH was evaluated for each breed and plotted against the autosomal chromosomal position of the SNP (Figure 5a–d). The thresholds for ES, HP, YL, and ZB cattle were 0.17, 0.31, 0.23, and 0.18, respectively. The results suggest that the occurrence of ROH exhibited significant variation across the genome among diverse cattle populations. In total, 30 hotspots in the top 1% of the studied population were identified (Table 1). Among these populations, HP cattle exhibited the highest number of identified ROH islands, followed by ES cattle, whereas ZB cattle demonstrated the lowest number of identified ROH islands. The highest numbers of SNPs and genes were observed in HP cattle, whereas ZB cattle had the lowest number of SNPs and genes. Furthermore, the shortest mean length of ROH was 57.1 Mb in ES cattle, whereas YL cattle exhibited the longest ROH length (105.32 Mb) and the greatest cumulative length (619.93 Mb).

**Figure 6:**
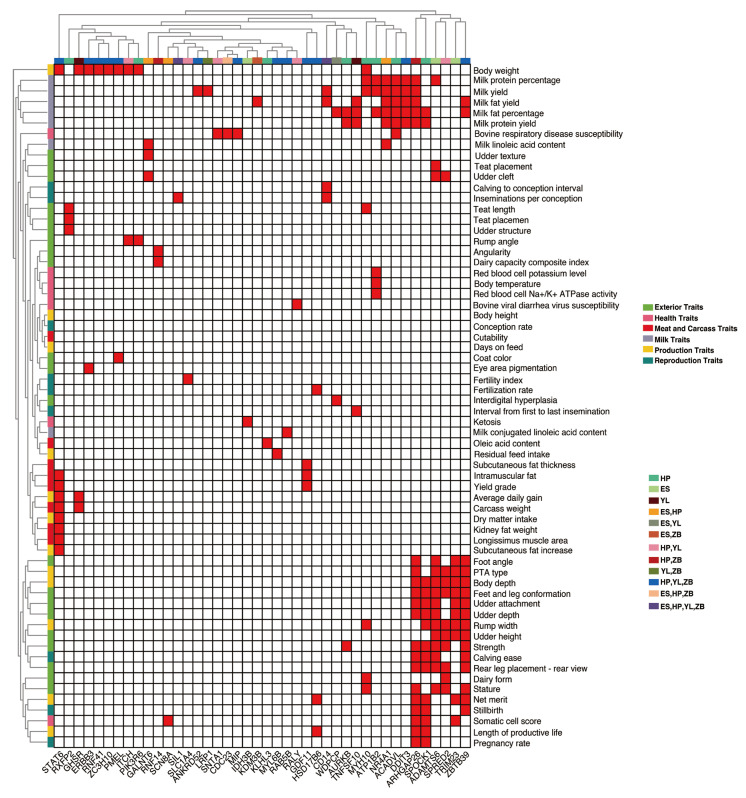
Cluster analyses of genes related to runs of homozygosity overlapping with QTLs in cattle. HP = Huangpi; YL = Yilin; ZB = Zaobei; ES = Enshi.

Identifying candidate genes in ROH islands facilitates the elucidation of ROH’s role in adaptation to economically important traits in livestock. In this study, we identified 475 unique genes across the four local breeds. To investigate the role of ROH-related genes in phenotypic traits, we predicted their potential functions using the cattle QTL database. The results showed that 42 ROH-related genes were associated with bovine QTLs associated with different traits (Figure 6). Interestingly, we observed that six genes—*ARHGAP26*, *SPOCK1*, *ADAMTS6*, *SPRED2*, *TRIM23*, and *ZBTB39*—clustered into one group that was mainly associated with exterior traits. Seven genes—*MY10*, *ATP1B2*, *NR4A1*, *ACADVL*, *DDIT3*, *ARHGAP26*, and *SPOCK1*—were found to be associated with milk traits. *STAT6* is associated with meat and carcass traits and production traits. Additionally, seven genes (*GHSR*, *ERBB3*, *RNF41*, *ZC3H10*, *PMEL*, *ITCH*, and *PIK3R6*) were associated with body weight. These results further revealed that genes overlapping with bovine QTL regions could be candidates affecting economically important traits in the local Hubei cattle population.

**Figure 7:**
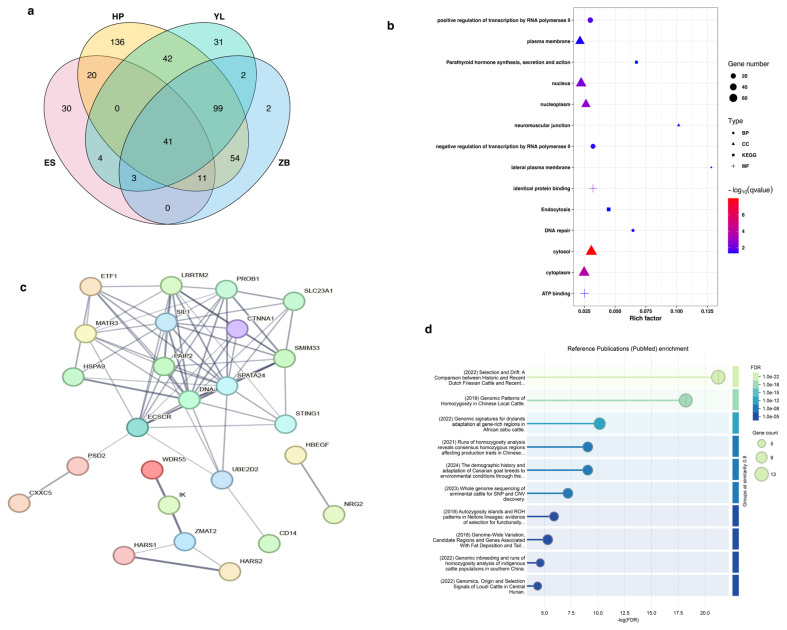
Venn diagram (**a**), functional enrichment (**b**), protein–protein interaction network (**c**), and reference publication enrichment (**d**) analyses of all shared genes under runs of homozygosity (ROH) islands. HP = Huangpi; YL = Yilin; ZB = Zaobei; ES = Enshi.

Furthermore, 41 shared ROH-related genes were observed among the studied breeds (Figure 7a). GO enrichment analysis revealed that these genes were mainly enriched in 12 terms (Figure 7b), including three biological processes (BP), seven cellular compositions (CC), and two molecular functions (MF). Notably, the significantly enriched terms for BP, CC, and MF were the positive regulation of transcription by RNA polymerase II, cytosol, and identical protein binding, respectively. KEGG enrichment analysis revealed that these genes were enriched in two pathways (Figure 7b). The significantly enriched pathway was endocytosis and parathyroid hormone synthesis, secretion, and action. These results suggest that these genes on the ROH islands may play a crucial role in economically important traits of cattle. Furthermore, we explored the protein–protein interaction relationship between shared ROH-related genes using the STRING database. The results showed that these genes clustered into three PPI networks (Figure 7c). The largest PPI network contained 18 genes, with several genes (*SIL1*, *CTNNA1*, *PAIP2*, *DNAJC18*, *SPATA24*, and *ECSCR*) displaying the highest node degrees. Notably, enrichment analysis of reference publications (PubMed) showed that most of these genes were associated with selection and drift in the Dutch Friesian and Holstein Friesian populations, as well as genomic patterns of homozygosity in local Chinese cattle (Figure 7d). These genes included *DNAJC18*, *PAIP2*, *SLC23A1*, *LRRTM2*, *SPATA24*, *SMIM33*, *SIL1*, *UBE2D2*, *MATR3*, *PROB1*, *STING1*, *CTNNA1*, and *ECSCR*. Interestingly, all the genes were located on bovine chromosome 7 (Table 2), with the region size of the ROH island varying by population.

## 4. Discussion

In this study, we successfully developed a 40K liquid capture chip specifically for indigenous Hubei beef cattle based on large-scale whole-genome resequencing data. The chip panel contained 42,686 potentially functional SNPs, including breed-specific, trait-related, and common sites found in the commercial chips. In addition, this chip exhibited a high SNP call rate among the local beef cattle breeds in Hubei. Furthermore, we demonstrated the effectiveness and utility of this SNP chip panel in beef cattle genetic research.

Performance analysis of this 40K chip panel revealed that a high SNP call rate was found in the studied population. We noted that both the SNP call rate and missingness rate varied among breeds. Notably, three breeds (ES, YL, and ZB) exhibited high SNP call rates (>99.5%), whereas the HP and YB breeds had a relatively low call rate. The observed discrepancy may be attributed to the heterogeneity in the breed-specific sites incorporated into the chip panel. Nevertheless, the SNP call rates within all studied breeds were over 99.0%, suggesting that this SNP chip panel has good genotyping performance. Furthermore, a comparative analysis between the 40K chip and commercially available bovine breeding chips revealed that 16,959 common loci were genotyped in the studied population, accounting for approximately 89.26% of the total common sites. These genotyped common sites had a higher missingness rate (0.212 ± 3.26) than those in the non-common sites (0.201 ± 2.85), whereas a lower heterozygote rate (30.8% ± 19.7) and MAF (0.214 ± 0.147) values were observed compared to those in the non-common sites (34.5% + 21.3 for heterozygote rate; 0.257 ± 0.157 for MAF). The results indicate that the SNP loci identified in this study, much like the common sites found in commercially available bovine breeding chips, demonstrate excellent capabilities in population genotyping. They can be effectively utilized in different studies on beef cattle population genetics, identifying selection signatures, and dissecting economically important traits.

We used the chip panel to determine population genetic characteristics between foreign and local beef cattle breeds, with 205 individuals representing six breeds. Both PCA and population structure analyses showed that the chip could effectively distinguish between foreign and local Hubei breeds. This may be due to the increase in gene heterogeneity, the expansion in genetic background, and the increase in genetic distance through long-term artificial breeding and natural selection. In contrast, this chip is slightly inferior in its ability to distinguish local beef cattle breeds in Hubei. The reason for this is probably that the genetic differences between local Hubei breeds are relatively small, as the physical distance between these local breed resources is smaller, they may be affected by the same environmental factors, and there may also be gene exchange in local Hubei populations.

We used this chip to identify ROH and associated genes in the local Hubei cattle population. Our data showed that 2547 ROH and 30 hotspots in the top 1% were identified in the study population. Here, we found clear differences in the total number and length of ROH in diverse local Hubei cattle populations. This is consistent with the results of previous studies by Xu et al. [37] and Purfield et al. [40]. Furthermore, we annotated 475 unique genes within the ROH islands and predicted their potential functions in phenotypic traits based on the cattle QTL database. Our data showed that 42 ROH-related genes were associated with bovine QTLs involved in different traits. We found that several genes (*ARHGAP26*, *SPOCK1*, *ADAMTS6*, *SPRED2*, *TRIM23*, and *ZBTB39*) clustered into a single group. Notably, these genes have been reported to be associated with bovine exterior traits [41]. Furthermore, we found seven known genes that were associated with milk traits: *MY10* [42], *ATP1B2* [42], *NR4A1* [43], *ACADVL* [44], *DDIT3* [43], *ARHGAP26* [41], and *SPOCK1* [41]. In addition, the *STAT6* gene is known to be associated with meat and carcass traits, as well as production traits [45,46]. Notably, seven genes—*GHSR* [47], *ERBB3* [48], *RNF41* [48], *ZC3H10* [48], *PMEL* [48], *ITCH* [49], and *PIK3R6* [50]—were associated with body weight. These results further revealed that genes overlapping with bovine QTL regions could be candidates affecting economically important traits in cattle, indicating the potential power of the 40K chip panel.

We noticed that 41 shared ROH-related genes were identified in all four local beef cattle breeds. Among them, 13 genes—*DNAJC18*, *PAIP2*, *SLC23A1*, *LRRTM2*, *SPATA24*, *SMIM33*, *SIL1*, *UBE2D2*, *MATR3*, *PROB1*, *STING1*, *CTNNA1*, and *ECSCR*—were found to be located on bovine chromosome 7 between 48.54 and 54.77 Mb. This region encompassed 119 SNP loci, of which 31 were common sites and 88 were newly identified sites. Interestingly, Hulsegge et al. also found that the largest ROH island in the Dutch Friesian breed was located on chromosome 7 between 50.03 and 50.86 Mb [51]. This region exhibits concordance with the ROH islands documented for taurine and indicine cattle breeds by Sölkner et al. (2014) [52] for eight local Chinese cattle breeds reported by Xu et al. [37] and Alpine cattle breeds [53]. Among these genes, the *CTNNA1* gene has been reported to be associated with muscle development, skeletal muscle growth, and meat tenderness [54,55]. *DNAJC18* and *SIL1* were found to be associated with heat stress and immunity, respectively, in the indigenous cattle population in southern China [56]. *PAIP2* [57] and *LRRTM2* [58] have been demonstrated to be associated with the maturation of male germ cells and male fertility. The MATR3 gene has also been identified within the overlapping region and is associated with adipose tissue deposition in bovine species [59]. The *ECSCR* gene plays a role in the regulation of insulin sensitivity and susceptibility to obesity [60]. These findings suggest that the genomic region on chromosome 7 between 48.54 and 54.77 Mb represents a significant ROH island contributing to economically important traits in cattle populations.

## 5. Conclusions

A liquid-phase SNP chip panel consisting of 42,686 SNP loci that encompass the primary genetic diversity of indigenous beef cattle in Hubei was developed using WGS data. This chip panel is proven to be a valuable tool for conducting genetic analyses and implementing selective breeding programs for beef cattle populations in Hubei. Population genetics and ROH analysis based on the chip data elucidated the genetic distinctions between local and foreign breeds and identified genes strongly associated with economically important traits. Although some evidence from Cattle QTLdb has been documented, further validation is necessary to understand the impact and regulatory mechanisms of these identified genes in beef cattle traits.

The current findings not only augment the genetic resources of indigenous bovine populations but also provide a substantive foundation for future molecular breeding strategies and genetic enhancement initiatives.

## Figures and Tables

**Table 1:** Statistics for runs of homozygosity in Hubei cattle population.

Breed	Sample Number	ROH Number	SNP Number	Gene Number	Longest ROH (Mb)	Mean ROH (Mb)
ES	33	8	338	175	533.612	57.100
HP	39	9	487	617	106.031	57.807
YL	52	7	424	352	619.931	105.322
ZB	71	6	308	327	410.002	61.872

ROH = runs of homozygosity; SNP = single-nucleotide polymorphism.

**Table 2:** Characterization of genomic regions with shared runs of homozygosity (ROH) islands.

Breed	CHR	Start (bp)	End (bp)	Region (Kb)	Number of SNPs	Genes
HP	7	48,542,265	54,706,580	6164.316	103	*DNAJC18*, *PAIP2*, *SLC23A1*, *LRRTM2*, *SPATA24*, *SMIM33*, *SIL1*, *UBE2D2*, *MATR3*, *PROB1*, *STING1*, *CTNNA1*, *ECSCR*
ES	7	48,542,265	53,412,209	4869.945	78	*DNAJC18*, *PAIP2*, *SLC23A1*, *LRRTM2*, *SPATA24*, *SMIM33*, *SIL1*, *UBE2D2*, *MATR3*, *PROB1*, *STING1*, *CTNNA1*, *ECSCR*
YL	7	48,542,265	53,053,673	4511.409	74	*DNAJC18*, *PAIP2*, *SLC23A1*, *LRRTM2*, *SPATA24*, *SMIM33*, *SIL1*, *UBE2D2*, *MATR3*, *PROB1*, *STING1*, *CTNNA1*, *ECSCR*
ZB	7	48,542,265	54,768,354	6226.09	108	*DNAJC18*, *PAIP2*, *SLC23A1*, *LRRTM2*, *SPATA24*, *SMIM33*, *SIL1*, *UBE2D2*, *MATR3*, *PROB1*, *STING1*, *CTNNA1*, *ECSCR*

CHR = chromosome; ROH = runs of homozygosity; HP = Huangpi; ES = Enshi; YL = Yilin; ZB = Zaobei.

## Data Availability

The data that support the study findings are available from the authors upon request.

## Supplementary Materials

The following supporting information can be downloaded at https://www.mdpi.com/article/10.3390/ani15091346/s1. Table S1: Detailed information on the WGS dataset for 98 beef cattle individuals; Figure S1: Study design and workflow of 40K liquid capture chip for Hubei indigenous beef cattle.
