# Supplementary material for: Development and Application of a 40 K Liquid Capture Chip for Beef Cattle
Source: Animals (Basel). 2025 May 7;15(9):1346. doi: 10.3390/ani15091346 (PMC12071055; doi:10.3390/ani15091346)
Supplement: Supplementary file 1 [file animals-15-01346-s001.zip › animals-3578934-supplementary.pdf]

Supplementary Table S1

Detailed information on the WGS dataset for 98 beef cattle individuals

Sample ID	Breeds	Raw Reads Number	Clean Reads Number	Raw Bases(bp)	Clean Bases(bp)	Effective Rate(%)	Q20(%)	Q30(%)
58339	HP	530007096	529961536	79501064400	79027779572	99.4	98.07	93.79
59727	YB	531860678	531860420	79779101700	79364294568	99.48	99.25	96.9
64383	HP	502521300	502467384	75378195000	74722572720	99.13	98.17	94.05
45098	HP	532547142	532506950	79882071300	79213922434	99.16	98.01	93.62
59424	YB	437667804	437667634	65650170600	65313825830	99.49	99.21	96.72
59047	YL	538164706	538164488	80724705900	80298512770	99.47	99.22	96.75
57804	YL	496544164	496507582	74481624600	74040882632	99.41	98.47	95.06
59210	YL	405352496	405352312	60802874400	60383277314	99.31	99.26	96.92
59056	YB	478768682	478768334	71815302300	71367780618	99.38	99.16	96.15
57731	YL	444587244	444557002	66688086600	66182936082	99.24	98.17	94.03
59555	ZB	415244618	415223794	62286692700	61923919270	99.42	98.28	94.03
58855	ZB	431645816	431645466	64746872400	64156173436	99.09	98.99	95.39
64367	HP	550303256	550271320	82545488400	81890689040	99.21	98.42	94.84
59264	YB	388814322	388769036	58322148300	58010014528	99.46	98.28	94.49
59621	ES	525736400	525736180	78860460000	78319092968	99.31	99.21	96.72
64515	ZB	533148926	533101568	79972338900	79517189824	99.43	98.63	95.57
59090	YL	397965100	397935670	59694765000	59340558834	99.41	98.32	94.59
58573	HP	469725426	469725144	70458813900	70033696426	99.4	98.81	94.69
40278	ZB	394425734	394409046	59163860100	58757022948	99.31	98.58	95.4

41017	ZB	543366412	543312398	81504961800	80805013078	99.14	98.47	95.04
40806	HP	517093286	517051366	77563992900	77118799596	99.43	98.45	94.97
41020	HP	429985506	429985328	64497825900	64124314616	99.42	99.25	96.92
41766	ZB	469125438	469125054	70368815700	69861577764	99.28	99.05	95.69
40955	YL	489571946	489571576	73435791900	72959403432	99.35	99.12	95.94
42244	ZB	551449052	551385192	82717357800	81993588446	99.13	98.28	94.36
41416	HP	523089644	523047076	78463446600	77808479854	99.17	98.28	94.5
33228	ES	418072362	418046784	62710854300	62378240622	99.47	98.01	93.59
40093	ZB	417032414	417032216	62554862100	62140462766	99.34	99.19	96.56
57362	ZB	392015848	391991628	58802377200	58391375906	99.3	98.54	95.23
59711	HP	497462658	497462458	74619398700	74217233102	99.46	99.2	96.66
59386	ES	504521176	504472026	75678176400	75108176610	99.25	98.22	94.28
57774	HP	457981720	457981540	68697258000	68204218784	99.28	99.21	96.73
59686	ES	393125848	393101340	58968877200	58649925746	99.46	98.56	95.32
59724	ZB	392525810	392502752	58878871500	58528207194	99.4	98.36	94.33
59018	HP	432248506	432212302	64837275900	64511819000	99.5	98.35	94.59
59693	ZB	534119586	534097154	80117937900	79525855016	99.26	98.33	94.56
64552	YB	505802518	505802322	75870377700	75421401390	99.41	99.03	96
59655	ES	488377452	488349176	73256617800	72611236338	99.12	98.44	94.82
60263	ES	411514478	411474198	61727171700	61358086606	99.4	98.32	94.51
60025	HP	524068956	524024140	78610343400	78102131022	99.35	98.36	94.73
57479	ZB	427436964	427436842	64115544600	63683598486	99.33	99.14	96.47
59499	ES	400772004	400771790	60115800600	59747261904	99.39	98.95	95.21
59447	ES	487364368	487364162	73104655200	72681986258	99.42	98.8	94.94

59541	ZB	405250712	405209250	60787606800	60353427910	99.29	98.06	93.7
59659	ZB	419740744	419716634	62961111600	62521505636	99.3	98.18	93.49
59749	YB	405352704	405352460	60802905600	60408476866	99.35	99.19	96.28
64577	HP	412117364	412080778	61817604600	61391056244	99.31	98.09	93.91
60308	HP	401395606	401395464	60209340900	59869022200	99.43	99.18	96.54
60060	HP	560843180	560793038	84126477000	83544883342	99.31	98.46	95.09
59925	HP	402999830	402999634	60449974500	60105549320	99.43	99.15	96.4
59834	ES	433891330	433849234	65083699500	64782267576	99.54	98.56	95.29
59503	YL	559214838	559214636	83882225700	83391367584	99.41	99.25	96.88
64554	HP	453278324	453249376	67991748600	67703146434	99.58	98.56	94.94
57152	YB	399012100	398980588	59851815000	59283745290	99.05	98.65	95.3
59550	ZB	437392800	437392578	65608920000	65170100088	99.33	99.06	95.73
57806	HP	624612102	624551838	93691815300	92895908384	99.15	98.25	94.31
59429	ES	537688566	537645156	80653284900	80091339340	99.3	98.33	94.67
59925	YL	397925804	397895896	59688870600	59300610008	99.35	98.4	94.74
59167	YB	477966228	477966028	71694934200	71344422610	99.51	99.08	96.18
59535	ZB	446484732	446453100	66972709800	66561925114	99.39	98.49	95.1
60318	ES	382993238	382965344	57448985700	57111054334	99.41	98.37	94.76
57354	HP	467651726	467615952	70147758900	69494134722	99.07	98.46	95.07
59820	HP	405168864	405141878	60775329600	60441912690	99.45	98.23	94.11
59078	YL	566052882	566052666	84907932300	84381903554	99.38	99.19	96.66
59812	HP	458677236	458655280	68801585400	68210260064	99.14	98.34	94.55
58908	ZB	408590248	408558838	61288537200	60732237302	99.09	98.42	94.86
59704	YL	505762418	505730832	75864362700	75110242768	99.01	98.25	94.06

40126	HP	438438750	438410700	65765812500	65243207950	99.21	98.19	93.98
59448	ZB	507261348	507195180	76089202200	75239291728	98.88	98.54	95.23
57345	YB	411169616	411169420	61675442400	61283239754	99.36	99.08	96.22
59613	HP	407128860	407103320	61069329000	60760184580	99.49	98.54	95.21
57893	YL	546076172	546075940	81911425800	81328896114	99.29	99.22	96.75
59193	YL	460127962	460108886	69019194300	68612020150	99.41	98.29	94.41
59705	YL	526422894	526422650	78963434100	78437426040	99.33	99.23	96.84
59391	YL	531484332	531451780	79722649800	78899344392	98.97	98.28	94.36
59773	ES	408325828	408325496	61248874200	60818262424	99.3	99.2	96.32
58847	ZB	558499612	558468002	83774941800	83089037824	99.18	98.56	95.27
59474	YL	476359524	476334026	71453928600	70825628664	99.12	98.4	94.7
47253	YB	490549948	490498690	73582492200	72915432524	99.09	98.26	94.21
22582	HP	416681754	416645756	62502263100	62024155722	99.24	97.88	93.22
47713	YB	534146814	534146404	80122022100	79620934410	99.37	99.06	95.68
47255	ES	536043402	535993280	80406510300	79698298996	99.12	98.55	95.3
34001	YL	465662490	465623858	69849373500	69392776922	99.35	98.17	94.15
47267	YB	417388120	417387962	62608218000	62250396018	99.43	99.15	96.44
41462	ES	439392882	439347130	65908932300	65358291960	99.16	98.23	94.26
47333	YB	489125470	489100832	73368820500	72866438610	99.32	98.38	94.74
22604	HP	414607232	414606054	62191084800	61751398120	99.29	98.88	95.35
22634	HP	448538020	448502836	67280703000	66826722214	99.33	98.47	95.02
46892	HP	431120726	431081096	64668108900	64212691500	99.3	98.22	93.82
59589	YB	602233324	602206868	90334998600	89660302030	99.25	98.54	95.27
58876	YL	483917828	483880562	72587674200	72028269702	99.23	98.12	93.91

64341	YB	410559100	410558752	61583865000	61201066574	99.38	99.21	96.35
45344	YL	402564650	402526472	60384697500	60018186558	99.39	98.23	94.21
46720	YB	542577162	542505106	81386574300	80846473606	99.34	98.35	94.65
46408	YB	464385972	464350410	69657895800	69232986126	99.39	98.22	94.11
64321	YL	400536962	400505052	60080544300	59655174976	99.29	98.21	94.03
47258	YL	483358260	483314506	72503739000	71844748794	99.09	98.42	94.85
22620	YB	613833990	613748240	92075098500	91528201666	99.41	98.34	94.62

YL=Yilin; YB=Yunba; ES=Enshi; HP=Huangpi;ZB=zaobei

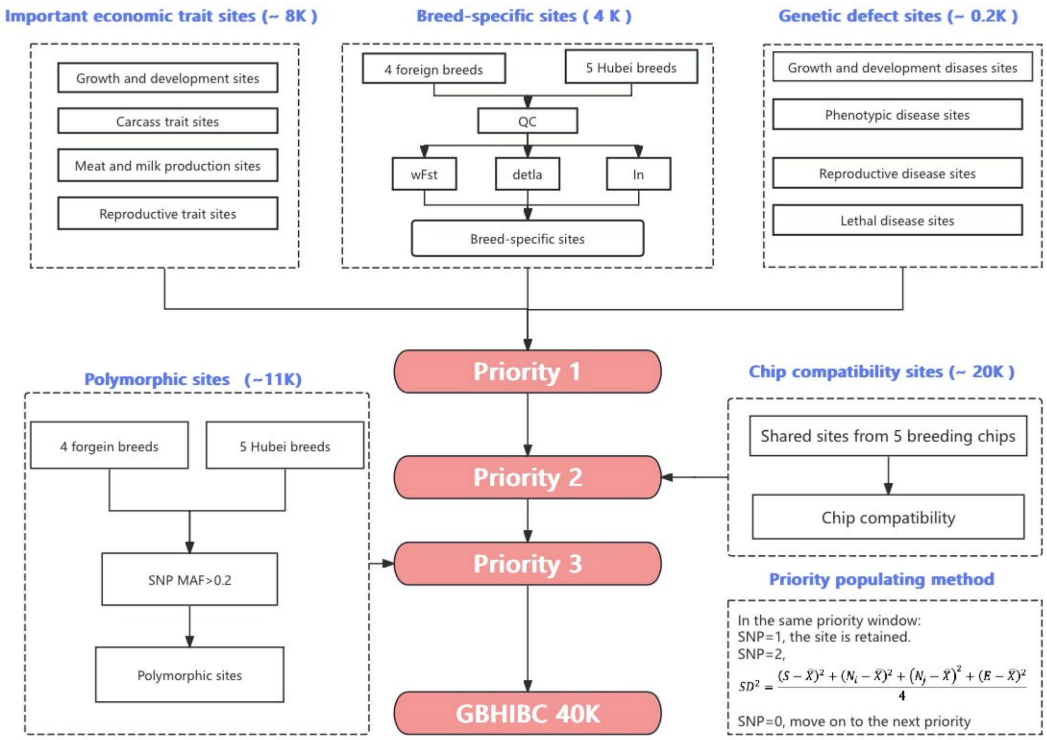

Supplementary Figure S1. Study design and workflow of 40K liquid capture chip for Hubei indigenous beef cattle.
